# Editorial: Microbiological Safety and Quality Aspects of Fermented Dairy Products

**DOI:** 10.3389/fmicb.2021.735560

**Published:** 2021-08-11

**Authors:** Juliano De Dea Lindner, Maria Cristina Dantas Vanetti, Baltasar Mayo, Uelinton Manoel Pinto

**Affiliations:** ^1^Food Technology and Bioprocess Research Group, Department of Food Science and Technology, Federal University of Santa Catarina (UFSC), Florianópolis, Brazil; ^2^Departament of Microbiology, Federal University of Viçosa (UFV), Viçosa, Brazil; ^3^Departament of Microbiology and Biochemistry, Instituto de Productos Lácteos de Asturias (IPLA), Consejo Superior de Investigaciones Científicas (CSIC), Villaviciosa, Spain; ^4^Department of Food and Experimental Nutrition, Food Research Center, Faculty of Pharmaceutical Sciences, University of São Paulo (USP), São Paulo, Brazil

**Keywords:** traditional cheeses, food safety, microbial interactions, biotechnology, omics

Fermented dairy products include a wide range of foods that are highly appreciated worldwide. The production processes, the type of milk, practices adopted during feeding, milking, and beyond can affect the quality and safety characteristics of these products. A vast array of microorganisms can be found in milk, and microbial succession during fermentation of multiple products, as well as during cheese ripening contributes to the desired properties of these foods. In addition to sensory and safety aspects, microorganisms present in fermented dairy foods can positively affect the health of people due to their potential probiotic nature and the production of beneficial metabolites such as vitamins, antioxidants, and antimicrobial compounds.

Despite recent innovations on microbial cultivation techniques, and the widespread use of a myriad of newly developed “omic” techniques, including genomic approaches through the use of High Throughput Sequencing in combination with advanced metabolomics, there are many dairy fermented products with limited information about their microbial composition (diversity) and dynamics (succession). This is particularly true for traditional regional products, which represent a rich niche for discoveries involving microorganisms with better conditions for recovery from disturbances and more reliable and robust for industrial production. The unknown microbiota can reveal new mechanisms of interactions and functions and new bioactive molecules with beneficial effects on the sensory aspect of food and on human health.

The goal of this Research Topic was to deepen the current knowledge in the microbial safety and quality aspects of fermented dairy products, gathering studies on the different types of products, practices, and how the microbiota affected the quality and safety attributes of these much-appreciated foods. This Research Topic brings a series of 14 articles related to the **microbiological safety and quality aspects of fermented dairy products**.

Two review articles provide important information for the cheese sector. The first presents an updated view on the diversity and safety of artisanal cheeses produced in Brazil, and the second addresses safety and technological issues regarding yeast contamination in white-brined cheeses. Pineda et al. tackle product diversity according to Brazilian geographical regions, the microbiological safety, and the huge challenges faced by the Brazilian artisanal cheese sector. The authors highlight that one of the main challenges is related to risks associated with foodborne pathogens when the quality of the raw milk is unsatisfactory. Artisanal cheese regulations have constantly been revised and adapted, considering the small-scale production of Brazilian artisanal cheeses, thus research collaborative efforts may help the sector to move toward better quality and safety goals. It is noteworthy that the economic income of thousands of rural families depends on artisanal cheese production in the country and there is an increased appreciation for artisanal cheese consumption in recent years. Geronikou et al. summarize the current knowledge on the identification of contaminant yeasts in white-brined cheeses, their occurrence and spoilage potential, interactions with other microorganisms, as well as guidelines to prevent cheese spoilage. The proliferation of spoilage yeast depends on ripening and storage conditions at each specific dairy, product characteristics, nutrients availability, and interactions with the microbial consortium, ultimately leading to decreased sensorial properties, shorter shelf life, and impaired quality. White-brined cheeses are worldwide produced with many different process flow charts. However, even small variations might have an impact on the parameters and, thereby, on the yeast species being able to proliferate. Detailed knowledge on variations on spoilage potential at strain level is still missing for this type of cheese. Furthermore, the acquisition of scientific knowledge about yeast interactions with other microorganisms related to milk and the cheese matrix will add to an optimized production of white-brined cheese of enhanced quality.

Milk is susceptible to contamination with pathogenic microorganisms. Microbial food safety along the dairy chain is an important topic, from public health and industry perspectives. The three papers submitted that dealt with food safety addressed issues related to the foodborne pathogen *Listeria monocytogenes*. This important microorganism can adapt and survive in food and food processing facilities where it can persist for years. The paper presented by Ricci et al. evaluated the heat resistance of *L. monocytogenes* in matrices involved in the production of Mozzarella di Bufala Campana Protected Designation of Origin (PDO) cheese. The 12 tested *L. monocytogenes* strains showed heterogeneous heat resistance profiles that ranged from 7 to <1 Log_10_ CFU/mL reductions after 8 min at 60°C. *D*-values and *z*-values, calculated for the most heat resistant strain, between 60 and 70°C were affected by the food matrix, being especially increased in drained cheese curd. As cheese curd stretching is not an isothermal process, a built secondary model was used to simulate microbial inactivation, and the authors were able to estimate the lethal effect of the process to be around 4 Log_10_ reductions. The data supplied may prove to be useful for Mozzarella cheese producers in determining appropriate time and temperature (kinetic parameters of heat inactivation) for producing fresh pasta filata cheese avoiding the presence of *L. monocytogenes* and improving microbial safety, efficacy, and sustainability of the process.

The paper carried by Bechtel and Gibbons analyzed the population genetic structure of 504 *L. monocytogenes* strains isolated from food with publicly available genome assemblies. The purpose of the study was to understand if genetically distinct populations are associated with particular foods. Using comparative and population genomic analysis, they identified genes that were present at a greater frequency in the population associated with cheese. The results suggest that particular *L. monocytogenes* genotypes may be associated with the colonization and persistence in certain food environments, such as dairy and cheese, and indicate potential candidate genes involved in the specialization to particular food substrates. This type of study can be very useful for tracking certain genotypes and to better understand food-borne outbreaks. Similarly, Mafuna et al. characterized 143 *L. monocytogenes* isolated in South Africa for their strain's genetic relatedness, virulence profiles, stress tolerance, and resistance genes. Examination of genes involved in adaptation and survival showed that some sequence types are well-adapted in food processing environments due to the significant over-representation of benzalkonium chloride resistance genes, stress tolerance genes, prophage (φ) profiles, plasmids profiles, and biofilm formation associated genes. The information provided in this study is important for a better understanding of the adaptation and survival of *L. monocytogenes* in the food-processing environments and the efficacy of using biocide disinfectants in facilities. Later in this editorial, a paper conducted in Italy by Cremonesi et al. will approach the effect of chlorine usage for sanitation procedures on the raw milk microbiota.

The next eight papers deal specifically with the analyses of the cheese microbiome. The composition of the microbiota has an important impact on the quality and safety of cheese due to the growth and interaction between microorganisms during processing and ripening. Therefore, much effort has been made by the authors to create molecular tools and omic systems to investigate the microbial community composition of cheese.

Dreier et al. developed a high-throughput quantitative real-time polymerase chain reaction (HT-qPCR) approach for rapid and cost-efficient quantification of microbial species in cheese. Preliminary results from model and downgraded commercial cheeses showed that the application of microfluidic HT-qPCR to complex fermented products could be of interest for identifying the microbial origin of quality defects. This new system is a promising approach that will allow, particularly in the production of raw milk cheese, simultaneous monitoring of the quality-relevant ripening microbiota, thereby opening up new perspectives for the control and assurance of high product quality. Afshari et al., Bottari et al., and Unno et al. used integrated molecular, analytical chemistry methods, and omics approaches to valuating the complexity of the microbiota and their metabolites involved in the cheese ripening.

The paper by Afshari et al. used bacterial 16S rRNA-gene sequencing, untargeted metabolomics, and data integration analyses to characterize and differentiate commercial Cheddar cheeses. Microbiota and metabolite compositions could be used to distinguish diverse time-ripened cheeses. Individual amino acids and carboxylic acids were positively correlated with the ripening age for some brands. The results suggest that multi-omics analyses have the potential to be used for discovering biomarkers for validating cheese age and brand authenticity. The paper by Bottari et al. used culture-dependent and independent microbial counts, high throughput 16S rRNA sequencing, length heterogeneity (LH-PCR), and ultra-performance liquid chromatography coupled to electrospray ionization tandem mass spectrometry (UPLC/ESI-MS) analysis to evaluate whether the composition of the bacterial community of Parmigiano Reggiano PDO cheese along with the specific peptide composition are more affected by the ripening times or by the cheese making process. The ripening time had the greatest impact on microbial dynamics and, consequently, on peptide composition. The potential use of peptides as markers of a specific microbial composition affords the possibility of taking advantage of it to protect and valorize the specificity and connection of traditional cheese to its production territory. The paper by Unno et al. integrated metagenomics using metagenomic amplicon sequencing and metabolomics with high-performance liquid chromatography (HPLC), and headspace gas chromatography mass spectrometry (HS-GC/MS) to reveal the microorganisms and components relationships in surface mold- and bacterial smear-ripened cheeses. Correlation analysis revealed that the abundance of specific bacteria was related to the formation of specific organic acids, free amino acids, and volatile compounds in these kinds of cheeses. The study will contribute to elucidate the role of non-starter cultures and possibly to select candidates for adjunct culture.

According to Johnson et al., one aspect of cheese quality that remains poorly understood is the variability of microbial subpopulations due to temporal or facility changes within production environments. In their work, the aim was to quantify the microbiome variability by measuring day-day and facility-facility changes. Microbial communities were characterized using 16S rRNA metabarcoding and by plating on selective growth media. Interestingly, facility differences were greater on food contact surfaces, whereas daily differences within each facility were mostly explained by variation in the milk and cheese. The results highlighted the complexity of the Cheddar cheese facility microbiome and demonstrated daily and facility-facility microbial variations, which might influence cheese quality.

The paper produced by Mancini et al. focused on the microbial and bacteriophages characterization from facilities that use natural whey starter (NWS) cultures for Trentingrana (Grana-like) PDO cheese production. Using culture-dependent methods and metataxonomic analysis, *Lactobacillus helveticus* was found occurring as the dominant and *Levilactobacillus brevis* as codominant NWS cultures. One hundred and twenty distinct phages were identified from 303 bacterial isolates from the NWS cultures. The authors found a possible correlation between bacterial starter biodiversity and the number of recovered lytic phages. They speculated that the presence of high biodiversity of NWS dairy bacteria biotypes is relevant to avoid phages dominance in NWS cultures, observing the recovery of a lower number of phages from the dairy plants with the higher biodiversity in *L. helveticus* biotypes. Phage predation can cause loss of NWS activity and generates a defective cheese. Also using the case of Trentingrana cheese, Cremonesi et al. evaluated the influence of chlorine products (sodium hypochlorite detergent) usage for sanitizing equipment on raw milk, NWS, and cheese microbiota and volatilome. Samples were subjected to culture-dependent and metagenomic analyses. Cheese volatilome was determined by solid-phase micro extraction-gas chromatography-mass spectrometry (SPME-GC-MS). As expected, a difference in microbial population related to chlorine usage in bulk milk, vat, and NWS samples was evidenced. The paper results support the idea that chlorine influences the milk and cheese microbiome and, consequently, cheese quality, safety, and sensory attributes. Furthermore, chlorine replacement is not associated with an increase of spoilage bacteria, staphylococci, and coliforms, but it leads to an increase in the milk microbial biodiversity. Further studies on sanitation strategies alternative to chlorine-based protocols are needed.

Cheese has been demonstrated to be an optimal carrier product to deliver viable probiotic bacteria. Pisano et al. produced probiotic Caciotta cheeses from pasteurized ewes' milk by using combinations of autochthonous microbial cultures, containing probiotic strains. It was demonstrated, using multivariate statistical analysis of ^1^H nuclear magnetic resonance spectroscopy-based metabolomics data, significant variations in the cheese' profiles both in terms of ripening time and strains combination when compared to a control cheese produced using commercial starter cultures. The data obtained have indicated the applicative potential of autochthonous lactic acid bacteria cultures, containing probiotic *Lactobacillus* and *Kluyveromyces* strains, for the production of potential functional cheese.

In the final paper, Perkins et al. combined culture-dependent techniques with whole-genome shotgun sequencing to light up the phylogenetic relationships among *Geotrichum candidum* and *Galactomyces* spp. strains of environmental and dairy origin. *G. candidum* is a yeast culture used on mold- and smear-ripened cheeses during ripening to promote the development of sensorial properties in the cheese surface. It was proposed a new multilocus sequence typing (MLST) scheme to optimally genotype isolates, substantially improving the knowledge on this culture to allow a better selection and control of *G. candidum* strains throughout the ripening.

## Author Contributions

All authors have made a substantial, direct and intellectual contribution to the work, and approved it for publication.

## Conflict of Interest

The authors declare that the research was conducted in the absence of any commercial or financial relationships that could be construed as a potential conflict of interest.

## Publisher's Note

All claims expressed in this article are solely those of the authors and do not necessarily represent those of their affiliated organizations, or those of the publisher, the editors and the reviewers. Any product that may be evaluated in this article, or claim that may be made by its manufacturer, is not guaranteed or endorsed by the publisher.

